# Distinguishing Between Gangrenous Cholecystitis and Ascending Cholangitis: A Case Study

**DOI:** 10.7759/cureus.28322

**Published:** 2022-08-23

**Authors:** Kenan Bakri, Kamil Abu-Shaban, Sishir Doddi, Xiaochen Liu, Garett A Begeman

**Affiliations:** 1 Radiology, University of Toledo College of Medicine, Toledo, USA; 2 Medical School, University of Toledo College of Medicine, Toledo, USA; 3 Radiology, University of Toledo Medical Center, Toledo, USA

**Keywords:** percutaneous cholecystostomy tube, cholangitis and endoscopic retrograde, acute cholangitis, gallbladder ultrasound, ascending cholangitis, gangrenous cholecystitis

## Abstract

Gangrenous cholecystitis is a potentially fatal complication of acute cholecystitis that presents with right upper quadrant pain and sepsis. Due to the overlap in clinical features with ascending cholangitis, gangrenous cholecystitis can be easily misdiagnosed, resulting in treatment delay. While the gold standard of diagnosis of gangrenous cholecystitis is direct visualization during surgery and tissue sampling to pathology, some imaging features can guide the diagnosis to appropriate early surgical treatment of gangrenous cholecystitis.

A 78-year-old female presented to the emergency department with right upper quadrant pain, sepsis, and altered mental status. Imaging findings on ultrasound and CT were suggestive of gangrenous cholecystitis. However, clinically the patient presented with ascending cholangitis symptoms. Instead of an emergent cholecystectomy, percutaneous cholecystostomy (PTC) was performed. After the PTC, the patient worsened clinically and despite surgical intervention, the patient expired due to septic shock and multiple organ failure.

## Introduction

Acute cholecystitis is inflammation of the gallbladder, usually secondary to blockage of the cystic duct or impaired gallbladder motility. Gangrenous cholecystitis (GC) is an uncommon but fatal complication of acute cholecystitis resulting in necrosis of the gallbladder wall [1]. The incidence of GC is more common in older patients and is associated with a higher white blood cell count (WBC) and diabetes [2]. The high fatality of GC is the result of a necrotic gallbladder wall which often leads to perforation [3]. Gallbladder wall ischemia results from vascular compromise and epithelial injury due to increased wall tension in an obstructed gallbladder. This compromise of the gallbladder wall eventually leads to translocation of bacteria into the bloodstream and septic shock [4]. Infection with gas-forming anaerobes, such as Clostridium perfringens, is associated with emphysematous cholecystitis, which is more likely to progress to GC and sepsis [5]. GC is difficult to diagnose pre-operatively, however, some studies have shown that computed tomography (CT) attenuation (Hounsfield unit, HU) can increase the sensitivity and specificity of CT in diagnosing GC. There are also pathognomonic ultrasound features. We present a case of GC on imaging pre-operatively and discuss how to differentiate it from acute cholecystitis and ascending cholangitis.

## Case presentation

A 78-year-old Caucasian female presented to the emergency department (ED) from an outside institution with sepsis and altered mental status. She had abdominal pain and weakness for a day and was admitted for fatigue and anemia two weeks prior. Past medical history included diabetes mellitus type 2 and breast cancer. In the ED, the patient was hypotensive (blood pressure 70/40 mmHg), febrile (101 °F), tachycardic (134 beats per minute), and tachypneic (40 respiratory rate). Patient was intermittently confused with mild right upper quadrant (RUQ) tenderness on palpation. The abdomen was nondistended. Extremities were cool with mottling. Labs showed leukocytosis, elevated creatinine, elevated liver enzymes, elevated total bilirubin, and lactic acidosis (Table 1). Blood culture was drawn. Patient was intubated in the ED and admitted to the ICU. She was started on vasopressors, broad spectrum antibiotics, and antifungals. Blood culture was later positive for Enterobacteriaceae which is commonly seen in acute cholangitis. Blood culture was also positive for C. perfringens which is common in elderly patients with acute cholangitis [6]. After the culture results, antibiotics were changed to piperacillin-tazobactam and clindamycin for a more focused coverage.

**Table 1:** Lab values on Presentation Labs on presentation included complete blood count, comprehensive metabolic panel, and lactate levels. The lab results showed evidence of leukocytosis, anemia, thrombocytopenia, lactic acidosis, elevated liver function tests, and acute kidney injury. BUN = Blood Urea Nitrogen; AST = Aspartate Transaminase; ALT = Alanine Transaminase * Indicates abnormal lab value

	Value	Reference Range
White Blood Cells	21,700*	4,000 - 11,000 /mm3
Hemoglobin	10.4*	11.7 - 15.5 g/dL
Hematocrit	30.6*	35 - 47 %
Platelets	122,000*	150,000 - 450,000 /mm3
Sodium	133*	134 - 146 mmol/L
Potassium	4.4	3.5 - 5.0 mmol/L
Chloride	100	98 - 109 mmol/L
CO_2_	16*	22 - 32 mmol/L
Anion Gap	17*	5 - 15 mmol/L
BUN	29*	5 - 27 mg/dL
Creatinine	1.76*	0.40 - 1.00 mg/dL
Baseline Creatinine	0.6	0.40 - 1.01 mg/dL
Alkaline Phosphatase	559*	39 - 130 U/L
AST	240*	0 - 41 U/L
ALT	78*	0 - 31 U/L
Total Bilirubin	4.6*	0.3 - 1.2 mg/dL
Direct bilirubin	2.4*	<0.3 mg/dL
Lactate	7.7*	0.4 - 2.0 mmol/L

**Figure 1:**
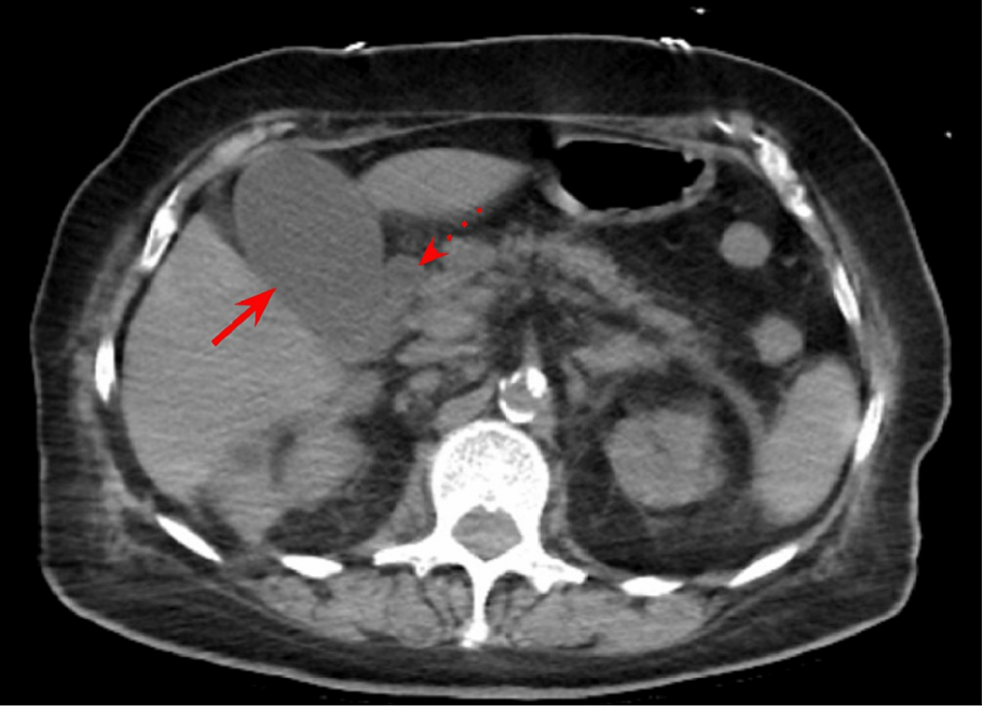
Initial CT abdomen and pelvis in the axial view CT abdomen and pelvis showed gallbladder distention (solid arrow) and increased prominence of the common bile duct (dashed arrow). No obstructing stone or mass.

**Figure 2:**
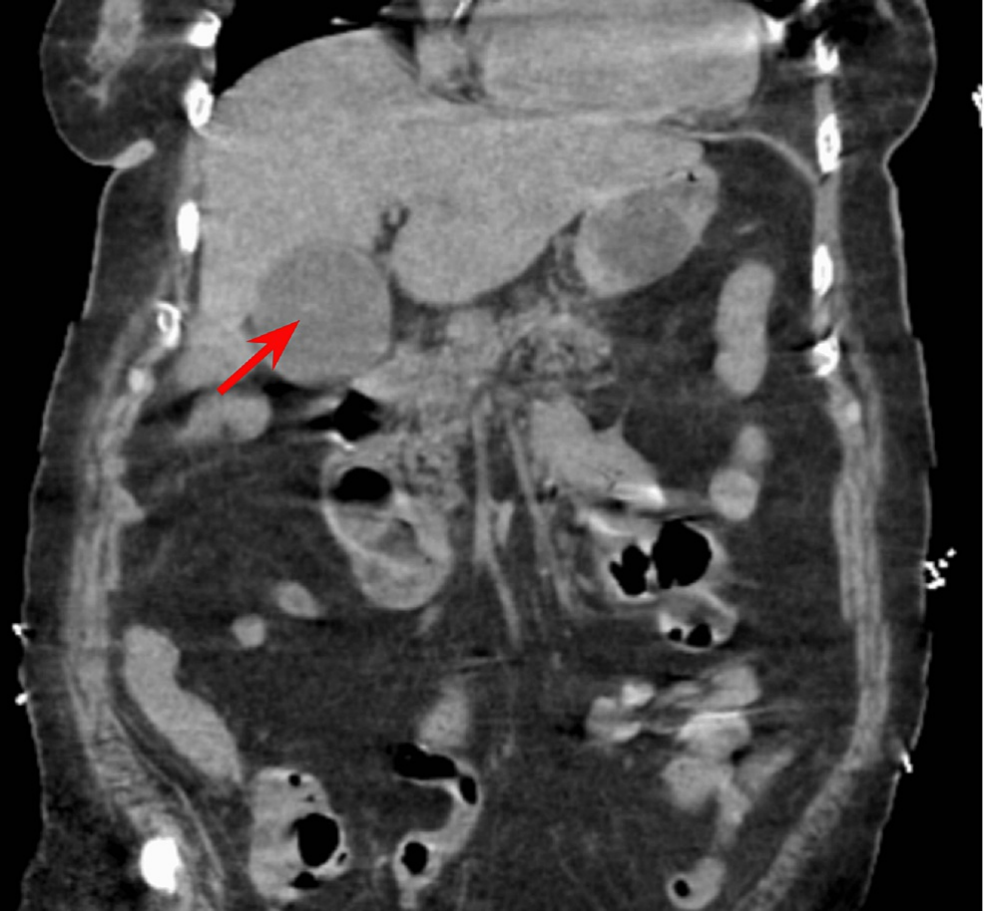
Initial CT abdomen and pelvis in the coronal view CT abdomen and pelvis in the coronal view with narrowing of the window shows intra-luminal membrane in the gallbladder (solid arrow).

**Figure 3:**
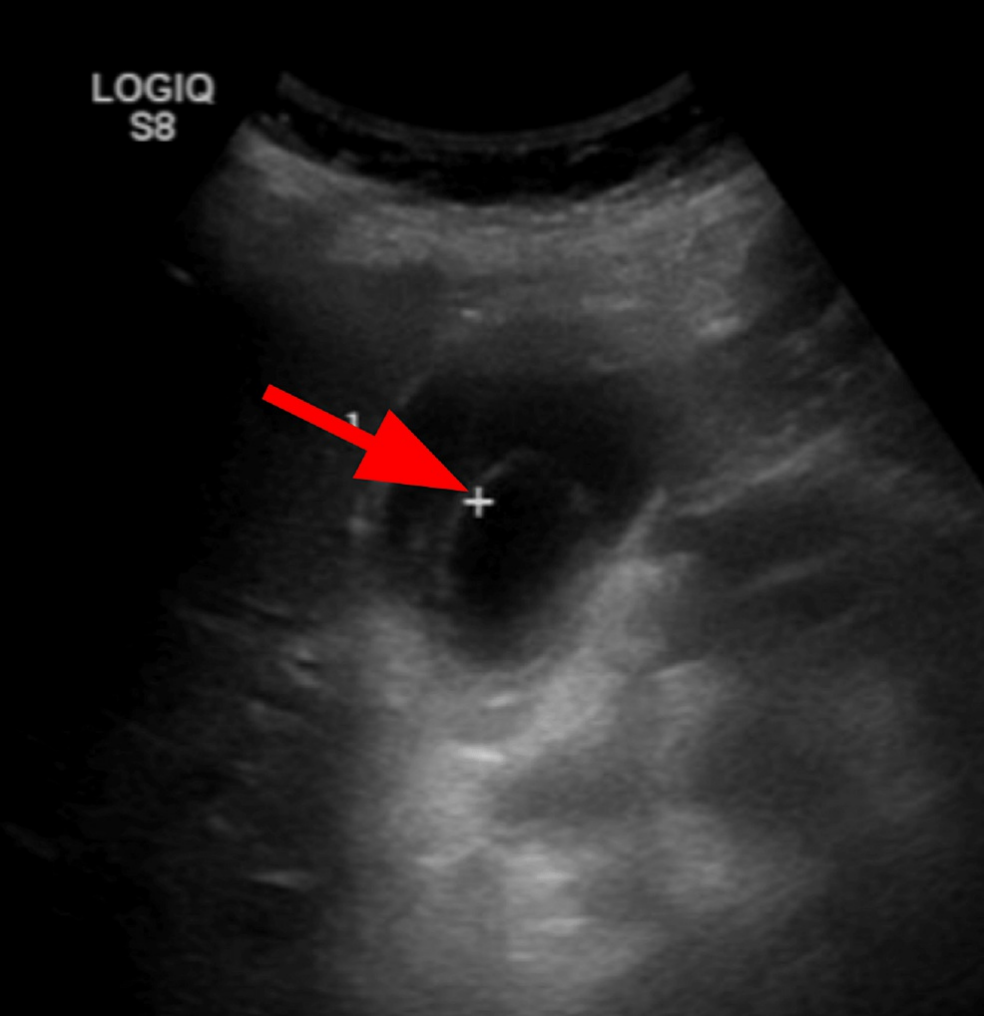
Right upper quadrant ultrasound Ultrasound showing delimitation of the gallbladder lumen, findings consistent with sloughed mucosa (solid arrow) seen in membranous gangrenous cholecystitis.

Initial chest x-ray (CXR) showed bilateral pulmonary infiltrates. Initial CT abdomen and pelvis without contrast showed retroperitoneal inflammation and lymphadenopathy, gallbladder distention, common bile duct prominence, and no obstructing gallstones (Figure 1). Narrowing the window/level of the non-contrast CT showed a subtle intra-luminal membrane of the gallbladder wall which was best seen on coronal view (Figure 2). Ultrasound showed sludge in the gallbladder with a round saclike structure within the gallbladder lumen (Figure 3). These findings were concerning for sloughed mucosa classically seen in membranous gangrenous cholecystitis. However, given the patient’s dilated common bile duct, elevated direct bilirubin, and elevated liver enzymes, the patient was clinically treated for ascending cholangitis. An urgent percutaneous cholecystostomy (PTC) tube was requested by the surgical team. At the time of PTC placement, there was a return of bile, bloody sloughed tissues, and clots. Following the PTC, the patient's condition continued to deteriorate and endoscopic retrograde cholangiopancreatography (ERCP) was performed. However, attempts to enter the bile duct failed and the patient was finally taken to the operating room for open cholecystectomy. The gallbladder was removed and there was evidence of intraperitoneal leak of infected bile and bloody necrotic gallbladder tissue during the operation. Intra-operatively, the gallbladder was grossly edematous without stones. The patient remained unstable on three vasopressors and unresponsive in the hours following the surgery. Following discussion with the family, the code status was changed to DNR-CC (do not resuscitate-comfort care) and the patient expired the same day.

## Discussion

Etiology and epidemiology

Gangrenous cholecystitis is the necrosis of the gallbladder wall due to associated inflammation with acute cholecystitis that causes marked gallbladder distention and vascular insufficiency [1,7]. Risk factors for gangrenous cholecystitis include cardiovascular disease and diabetes. Gangrenous cholecystitis can be distinguished from ascending cholangitis by careful clinical evaluation in combination with imaging and laboratory findings. GC occurs in 2 to 20% of acute cholecystitis cases and is a severe complication with a high mortality. A 2015 study by Önder et al. found a 17.8% mortality rate, but the rate may be as high as 50% in patients with comorbidities [3]. A study by Merriam et al. found that compared to non-gangrenous cholecystitis, which is often associated with middle-aged females, GC was more likely to develop in patients of older age (>50 years), male gender, diabetes, and a history of cardiovascular disease [8]. Given our patient’s age and medical history of diabetes and hypertension, she had an elevated risk of GC.

Diagnostic criteria - labs and imaging

Gangrenous cholecystitis is difficult to diagnose pre-operatively, which is why surgical management for these patients is often delayed [4]. There are certain labs and imaging findings, however, that should increase suspicion for GC given the right clinical context. In a study of 75 cases with different gallbladder pathologies, the most specific signs for GC on CT of the abdomen were gas in the wall or lumen (100%), intraluminal membranes (99.5%), irregular or absent wall (97.6%), and abscess (96.6%) [2]. Similar to gangrenous necrosis of the bowel, the growth of bacteria with the release of toxins and necrosis of the gallbladder wall can cause an increase in attenuation on CT. A study by Sureka et al. found that a combined unenhanced CT density of the gallbladder wall greater than 31.5 HU and intraluminal bile greater than 12.5 HU could predict GC with a high sensitivity and specificity [8]. On ultrasound, findings pointing towards GC include decreased focal wall perfusion on color doppler, irregular mucosal outline, gallbladder wall thickening and delamination, gas within the gallbladder, absence of gallstones, and large pericholecystic collections [9]. In terms of non-imaging findings, Merriam et al. found that 58% of cholecystitis patients with WBC count greater than 17,000/mL and 83% with WBC count greater than 20,000/mL had GC [10]. Fagan et al. also found that a WBC count greater than 15,000/mL was consistent with GC as opposed to non-GC [4]. Our patient’s WBC count was 21,700/mL.

Comparison and treatment

The similar clinical presentation between ascending cholangitis and gangrenous cholecystitis often makes them difficult to differentiate. Our patient presented with right upper quadrant pain, hypotension, fever, elevated bilirubin, and elevated liver enzymes, which strongly suggests a diagnosis of ascending cholangitis. However, patients who are septic due to GC can also present with similar symptoms, such as the presenting patient [11]. It is crucial to distinguish between ascending cholangitis and gangrenous cholecystitis due to the differences in management. The gold standard treatment for gangrenous cholecystitis is urgent cholecystectomy [3]. Operative treatment for ascending cholangitis is urgent biliary drainage [12]. 

Misdiagnosing GC for ascending cholangitis was an unfortunate event that caused a delay in appropriate surgical treatment for the presenting patient, who eventually expired from septic shock. In retrospect, the ideal management for this patient would have been an urgent cholecystectomy from the beginning rather than biliary drainage. The necrotic gallbladder wall was unable to hold the PTC resulting in perforation of the fragile gallbladder and leakage of necrotic content. The ERCP increased the patient’s risk of infection and further delayed the much-needed cholecystectomy.

## Conclusions

Gangrenous cholecystitis is a fatal complication of acute cholecystitis that occurs due to infarction of the gallbladder wall and can lead to sepsis. It is crucial to distinguish gangrenous cholecystitis from ascending cholangitis and uncomplicated acute cholecystitis as they have differing management. Some key features that point towards gangrenous cholecystitis include intra-luminal membranes/delamination of the gallbladder wall, gas in the gallbladder wall, and a decreased gallbladder wall perfusion on color doppler.
